# Genetic Analysis of 28 Chinese Families With Tyrosinase-Positive Oculocutaneous Albinism

**DOI:** 10.3389/fgene.2021.715437

**Published:** 2021-10-11

**Authors:** Linya Ma, Jianjian Zhu, Jing Wang, Yazhou Huang, Jibo Zhang, Chao Wang, Yuan Zhou, Dan Peng

**Affiliations:** ^1^Department of Medical Genetics, Changde First People’s Hospital, Changde, China; ^2^Changsha Kingmed Center for Clinical Laboratory, Changsha, China; ^3^Affiliated Hospital of Changde City, University of South China, Hengyang, China

**Keywords:** *OCA2 gene*, missense variants, oculocutaneous albinism, next-generation sequencing, novel variants

## Abstract

**Background:**

Tyrosinase-positive oculocutaneous albinism (OCA, type II, OCA2) is an autosomal recessive genetic disease in which the biosynthesis of melanin decreases in the skin, hair, and eyes. OCA2 disease is caused by mutations in *OCA2* gene. The gene product plays a role in regulating the pH of melanosomes. Up to now, hundreds of *OCA2* mutations have been reported and novel variants are still being discovered.

**Methods:**

In this study, we reviewed the records of OCA2 patients who had conducted albinism genetic testing, and then analyzed the clinical and genetic information of 28 OCA2 patients who had been genetically diagnosed by using Sanger sequencing and next-generation sequencing.

**Results:**

In this study, we reported 31 variants screened from 28 Chinese OCA2 families, and characterized the detailed molecular and clinical presentations. There were 12 novel variants among all detected variants, including 3 missense variants (p.G393V, p.T482A, and p.R720P), 4 frameshift variants (p.R53Gfs^∗^49, p.N279Kfs^∗^17, p.I469Lfs^∗^4, p.I655Nfs^∗^12), 2 splicing variants (c.1637-2A > G, c.1951 + 1G > C), 2 stopgain variants (p.L278X, p.W652X) and 1 insertion variants (p.P315LinsT). One potential cluster of missense variants was implicated indicating the important roles of the underlying domains in OCA2 pathogenesis.

**Conclusion:**

Our results were beneficial for diagnosis and precision clinical management for *OCA2*-related disorder, and this study expanded the mutation spectrum of oculocutaneous albinism.

## Introduction

Albinism is a heterogeneous genetic disorder in which a group of genes related to pigment synthesis have mutated, leading to melanin deficiency. According to its clinical symptoms, it can be divided into oculocutaneous albinism (OCA) and ocular albinism (OA). Oculocutaneous albinism is a crowd of inherited disorders of melanin biosynthesis characterized by a generalized reduction in pigmentation of hair, skin and eyes (Gronskov et al., 2007), whereas in OA patients only the ocular pigment is deficient. The degree of hypopigmentation of skin and hair varies with the types of OCA. Oculocutaneous albinism is classified into non-syndromic oculocutaneous albinism and syndrome oculocutaneous albinism based on symptoms such as the presence of bleeding diathesis, immunodeficiency and neurological dysfunction (Simeonov et al., 2013). Mutations in *TYR*, *OCA2*, *TYRP1*, *SLC45A2*, *SLC24A5*, and *LRMDA* lead to six different non-syndromic OCA subtypes, they are OCA1, OCA2, OCA3, OCA4, OCA6, and OCA7, respectively (Arveiler et al., 2017). The prevalence of all forms of albinism varies considerably worldwide, estimated at approximately 1/17000, the mutation carrier rate is about 1 in 65 (Montoliu et al., 2014).

Tyrosinase-positive oculocutaneous albinism (OCA, type II, OMIM 203200) is an autosomal recessive genetic disease that reduces the biosynthesis of melanin in the skin, hair and eyes. Patients with OCA2 have characteristic visual abnormalities associated with albinism, including decreased vision and nystagmus. These symptoms are usually milder than those with OCA1 (Lee et al., 1994). OCA2 was first identified in 1993, in a patient with tyrosinase-positive oculocutaneous albinism. According to epidemiological survey, OCA2 is the most prevalent subtype in Africa and accounts for 30% of OCA cases worldwide. Studies have revealed that OCA2 is caused by mutations in *OCA2*, the human cDNA contains 3143 bases which encodes a protein that corresponds to the “pink-eyed dilution” (p) mouse mutant. In addition, the gene product plays a role in regulating the pH of melanosomes (Yuasa et al., 2007). Up to now, lots of *OCA2* mutations have been reported and novel variants are still being discovered (Maurano et al., 2012; Martinez-Garcia and Montoliu, 2013; Wang et al., 2016; Qiu et al., 2018; Lin et al., 2019; Luo et al., 2019; Yang et al., 2019; Zhong et al., 2019; Chuan et al., 2021; Xu et al., 2021). In recent years, multiple groups of researchers have conducted epidemiological surveys on Chinese albinism patients. Although the proportions of each type are different, OCA1 is recognized as the main type of albinism, and exons 1 and 2 are mutation hotspots. The proportions of OCA2, OCA4, HPS1 and unknown mutations are behind (Lin et al., 2019; Luo et al., 2019; Chuan et al., 2021; Xu et al., 2021).

In this study, we performed mutational screening of OCA2 in 28 Chinese OCA2 patients. After literature search and database retrieval, 12 novel variants in OCA2 were identified. Our results provide new insight into the underlying mechanisms of OCA2 and at the same time, provide important information for genetic testing and counseling.

## Materials and Methods

### Patients

We reviewed the records of patients undergoing genetic testing for albinism in the Changde First People’s Hospital and Changsha Kingmed Center for Clinical Laboratory in recent years. We recruited 28 OCA2 families from Hunan Province, China. Among the 28 families, there are 28 patients, including 15 females and 13 males. All patients denied family history of consanguinity. These patients all have obvious albinism phenotype, but due to the incompletely medical records, the clinical data did not record their skin color, hair color, iris color and ophthalmic phenotype in detail.

### Ethics Statement

A written informed consent was obtained from each subject or their guardians to participate in this study. The study was conducted according to the guidelines of the Declaration of Helsinki, and approved by the Ethics Committee of Changde First People’s Hospital (protocol code 2020-014-01) and approved on January 23, 2020.

### Genomic DNA Preparation and Next-Generation Sequencing

Genomic DNA was extracted using whole blood DNA extraction kit (Tiangen Biotechnology, Beijing, China). The library was prepared by randomly fragmenting 2 μg of genomic DNA into fragments by ultrasonic shearing. According to the standard Illumina protocol, a HiSeq2500 sequencer was used for sample sequencing. The amplified DNA was captured with a Genodermatosis-related Gene Panel, which can capture all exons and splicing sites of more than 100 related genes. The raw reads were filtered to obtain high-quality clean reads, and mapped to the human reference genome (UCSC hg19), and the sention software suite was used to call Single Nucleotide Variants (SNVs) and small insertions or deletions (InDels).

### Validation of Variants and Inheritance Analysis

All candidate pathogenic variants were verified by Sanger sequencing in the reported families to verify the heritability of the variants. We designed specific primers (Supplementary Table 1) to amplify the region containing the corresponding variation by polymerase chain reaction (PCR). The PCR products were then sequenced on ABI 3730XL Genetic Analyzer (Applied Biosystems Life Technologies) according to manufacturer s protocols.

### Variant Classification and *in silico* Analysis

The data obtained by sequencing was screened according to the following criteria. First, the variants with minor allele frequency (MAF) >0.01 in the following three SNP databases were excluded. Including gnomAD,^1^ 1000 Genome project,^2^ dbSNP^3^ and ESP6500.^4^ Second, we used the ClinVar database,^5^ the albinism database,^6^ the human gene mutation database (HGMD^7^) and OMIM^8^ to annotate variants. Third, in order to determine the pathogenicity of novel mutations, we *in silico* analyzed the pathogenicity of novel mutations with various tools, which included the programs of Sorting Intolerant From Tolerant (SIFT^9^), Polymorphism Phenotyping v2 (PolyPhen2^10^), Mutation Assessor,^11^ Protein Variation Effect Analyzer (PROVEAN^12^) and CADD.^13^ Subsequently, we used I Mutant2.0^14^ to evaluate the protein stability changes upon novel variants and clustalX2 for the protein conservation analysis. The structure changes of protein caused by amino acid substitutions were simulated by I-TASSER.^15^ Finally, according to the American Medical Genetics and Genomics (ACMG) guidelines, all detected novel variants were classified into pathogenic, likely pathogenic, uncertain significance, and likely benign or benign.

## Results

### Clinical Manifestation

By reviewing the records of patients who have performed albinism genetic testing in the First People’s Hospital of Changde and Changsha Kingmed Center for Clinical Laboratory in recent years, the clinical information of 28 patients who had been diagnosed as OCA2 through genetic testing was collected. All patients had typical OCA symptoms, including varying degrees of hypopigmentation on the skin, hair and iris. Moreover, patients did not show any other symptoms involving other systems. Among 28 patients, only 7 parents had tested for albinism-related genes (Supplementary Table 1). The clinical characteristics and mutant alleles of these 28 patients were shown in Table 1.

**TABLE 1:** Clinical characteristics and genotypes of the 28 patients.

Patient ID	Gender	Age	Molecular diagnosis	Mutations			
				**Allele 1**	**Parental**	**Allele 2**	**Parental**
1	M	25	OCA2	c.1178G > T	Ma	c.1178G > T	Pa
2	F	5	OCA2	c.1963dupA	Ma	c.1444A > G	Pa
3	F	9	OCA2	c.1255C > T	Ma	c.1255C > T	Pa
4	M	3	OCA2	c.833T > G	NA	c.406C > T	NA
5	F	1	OCA2	c.1349C > T	NA	c.1349C > T	NA
6	M	10	OCA2	c.2228C > T	NA	c.593C > T	NA
7	F	9	OCA2	c.1441G > A	NA	c.2159G > C	NA
8	F	14	OCA2	c.2344G > A	NA	c.1844A > G	NA
9	F	6	OCA2	c.406C > T	NA	c.1955G > A	NA
10	M	4	OCA2	c.1663C > T	NA	c.2330G > A	NA
11	M	4	OCA2	c.1349C > T	NA	c.1441G > A	NA
12	M	24	OCA2	c.1255C > T	Ma	c.2180T > C	Pa
13	F	5	OCA2	c.1182 + 1G > A	NA	c.1405_1406delATinsC	NA
14	F	8	OCA2	c.1255C > T	NA	c.2323G > C	NA
15	F	26	OCA2	c.1182 + 1G > A	NA	c.1405_1406delATinsC	NA
16	F	25	OCA2	c.156delC	NA	c.1441G > A	NA
17	F	17	OCA2	c.1255C > T	NA	c.1349C > T	NA
18	F	3	OCA2	c.1255C > T	NA	c.2180T > C	NA
19	M	13	OCA2	c.163delG	NA	c.1441G > A	NA
20	F	22	OCA2	c.1441G > A	NA	c.2344G > A	NA
21	M	5	OCA2	c.808-3C > G	NA	c.1441G > A	NA
22	M	21	OCA2	c.1951 + 1G > C	NA	c.1423A > G	NA
23	F	9	OCA2	c.2359G > A	NA	c.1255C > T	NA
24	M	2	OCA2	c.1363A > G	NA	c.1637-2A > G	NA
25	F	7	OCA2	c.593C > T	NA	c.2228C > T	NA
26	M	4	OCA2	c.406C > T	Ma	c.593C > T	Pa
27	M	2	OCA2	c.2323G > A	Ma	c.830_836dup	Pa
28	M	1	OCA2	c.944_945insCAC	Ma	c.1139_1141del	Pa

### Mutation Pattern and Potential Missense Variant Clusters in *OCA2*

**FIGURE 1:**
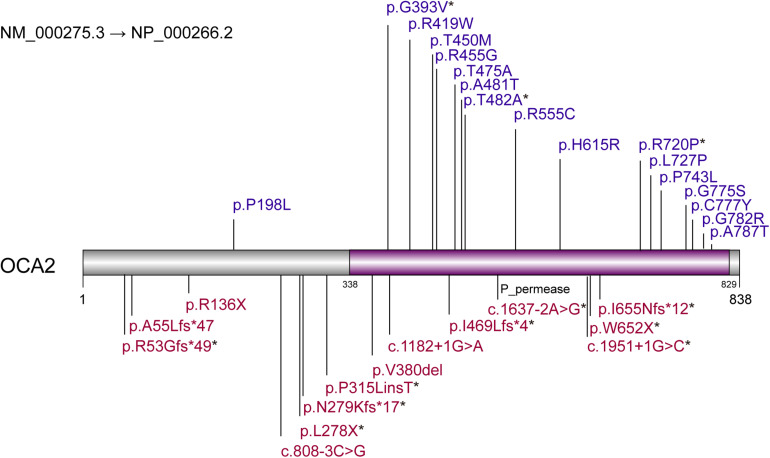
Variants pattern of *OCA2*. Loss-of-function variants are labeled above the diagram. Missense variants are labeled below the diagram. Purple region represents P-permease domain. * represents the novel variants.

Based on the analysis and statistics of the sequencing results, 2 of the 28 patients were single homozygous (Patient 1 and 3 in Table 1), and the rest 26 probands were compound heterozygous. Moreover, missense variants (54.8%, 17/31) were the most prevalent mutation. To investigate the mutation pattern of *OCA2*, we aggregated the mutations information of all patients. There were 31 variants in 28 patients, including twelve likely gene-disrupting (LGD) variants (6 frameshift, 2 stopgain, 4 splice-site), 1 deletion,1 insertion and seventeen missense variants (Table 2 and Figure 1). We observed one potential missense cluster, 16 out of 17 missense variants were clustered in P-permease domain (Figure 1).

**TABLE 2:** Summary of OCA2 variants identified in this study.

Nt change	gDNA change(chr15)	Exon No.	AA change	Function	Minor Allele frequency (gnomAD)	Mutation frequency
c.156delC	g.28326865delG	Exon2	p.R53Gfs*49	frameshift	0.00001217	1/56
c.163delG	g.28326858delC	Exon2	p.A55Lfs*47	frameshift	0.00001079	1/56
c.406C > T	g.28273126G > A	Exon4	p.R136X	stopgain	0.00001591	3/56
c.593C > T	g.28267700G > A	Exon6	p.P198L	missense	0.0001238	3/56
c.808-3C > G	g.28261332-3G > C	Intron7	–	splicing	0.000003977	1/56
c.833T > G	g.28261307A > C	Exon8	p.L278X	frameshift	–	1/56
c.830_836dup	g.28261304_g.28261310dup	Exon8	p.N279Kfs*17	frameshift	–	1/56
c.944_945insCAC	g.28260021_g.28260022insGTG	Exon9	p.P315LinsT	insertion	–	1/56
c.1139_1141del	g.28234788_g.28234790delCCA	Exon11	p.V380del	deletion	–	1/56
c.1178G > T	g.28234751C > A	Exon11	p.G393V	missense	0.000003979	1/56
c.1182 + 1G > A	g.28230320 + 1C > T	Intron11	–	splicing	–	2/56
c.1255C > T	g.28230319G > A	Exon13	p.R419W	missense	0.0002659	6/56
c.1349C > T	g.28230225G > A	Exon13	p.T450M	missense	0.00001776	3/56
c.1363A > G	g.28230211T > C	Exon13	p.R455G	missense	0.0002453	1/56
c.1405_1406delATinsC	g.28228588_g.28228589delinsG	Exon14	p.I469Lfs*4	frameshift	–	2/56
c.1423A > G	g.28228571T > C	Exon14	p.T475A	missense	–	1/56
c.1441G > A	g.28228553C > T	Exon14	p.A481T	missense	0.008427	6/56
c.1444A > G	g.28228550T > C	Exon14	p.T482A	missense	–	1/56
c.1637-2A > G	g.28202881-2T > C	Intron15	–	splicing	–	1/56
c.1663C > T	g.28202855G > A	Exon16	p.R555C	missense	0.0001186	1/56
c.1844A > G	g.28197037T > C	Exon18	p.H615R	missense	0.04345	1/56
c.1951 + 1G > C	g.28196930 + 1C > G	Intron18	–	splicing	0.000003984	1/56
c.1955G > A	g.28171397C > 7	Exon19	p.W652X	stopgain	–	1/56
c.1963dupA	g.28171390dupT	Exon14	p.I655Nfs*12	frameshift	–	1/56
c.2159G > C	g.28116385C > G	Exon21	p.R720P	missense	–	1/56
c.2180T > C	g.28116364A > G	Exon21	p.L727P	missense	–	2/56
c.2228C > T	g.28116316G > A	Exon21	p.P743L	missense	0.0001344	2/56
c.2323G > A	g.28096543C > T	Exon22	p.G775S	missense	0.000008028	2/56
c.2330G > A	g.28096536C > T	Exon22	p.C777Y	missense	0.00003277	1/56
c.2344G > A	g.28090193C > T	Exon23	p.G782R	missense	0.000007071	2/56
c.2359G > A	g.28090178C > T	Exon23	p.A787T	missense	0.00004374	1/56

### Pathogenic Novel Variant Pattern of *OCA2*-Related OCA2 Genes

**FIGURE 2:**
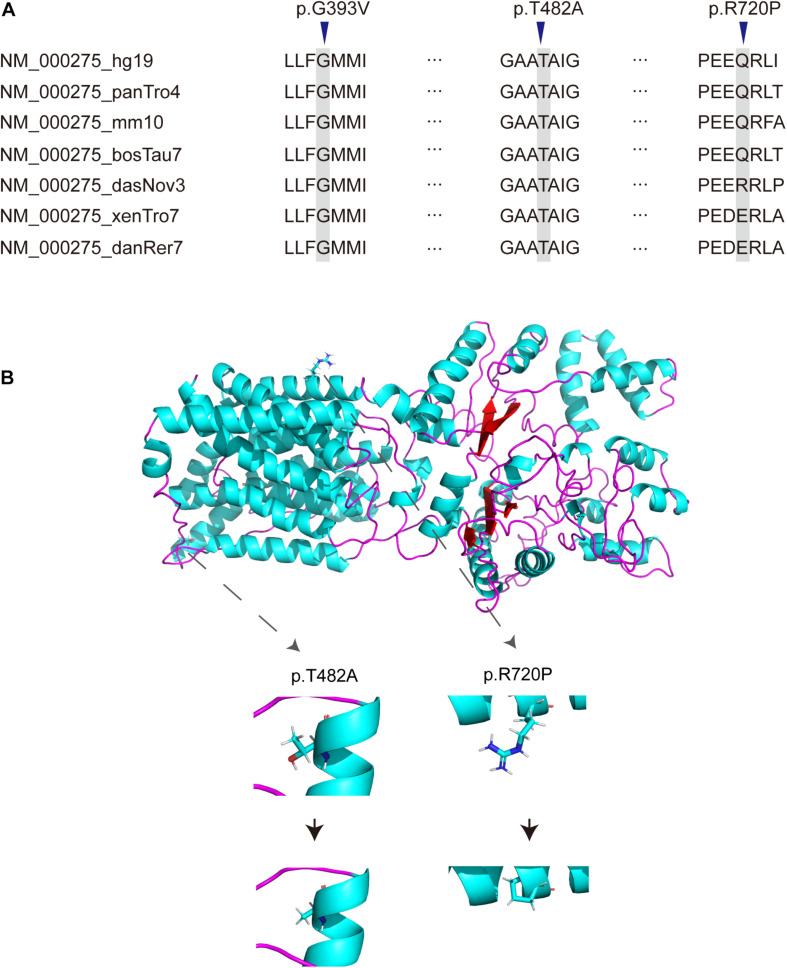
Novel missense variants of *OCA2*. **(A)** Conservation analysis of *OCA2* missense variants. **(B)** Simulation of the amino acids conformation changes by I-TASSER.

There were 12 novel variants among all variants, including 3 missense variants. Of the remaining 19 reported mutations, six were reported for the first time in the Chinese population, including p.A55Lfs^∗^47, p.L727P, p.P743L, p.G775S, p.C777Y, and p.G782R. By comparing the alignments of OCA2 orthologous peptide sequences of the three novel missense variants (p.G393V, p.T482A, p.R720P), most variants show evolutionary conservation (Figure 2A) and predicted to be damaging or probably damaging by PolyPhen2, PROVEAN, SIFT and had a high CADD and Mutation Assessor score (Table 3). The p.T482A and p.R720P were assessed to decrease the OCA2 protein stability by I Mutant2.0, which was consistent with the simulated result by I-TASSER that amino acid substitutions resulted in the structure changes of protein (Figure 2B).

**TABLE 3:** Pathogenicity analysis of novel variants.

Nt change	AA change	SIFT	Polyphen2	Mutation assessor	PROVEAN	CADD score	Variant classification	Evidence criterion
c.156delC	p.R53Gfs*49	–	–	–	–	–	Pathogenic	PVS1 + PM2 + PP3
c.830_836dup	p.N279Kfs*17	–	–	–	–	–	Pathogenic	PVS1 + PM2 + PP3
c.1405_1406delATinsC	p.I469Lfs*4	–	–	–	–	–	Pathogenic	PVS1 + PM2 + PP3
c.1963dupA	p.I655Nfs*12	–	–	–	–	–	Likely pathogenic	PVS1 + PM2
c.1637-2A > G	c.1637-2A > G	–	–	–	–	–	Pathogenic	PVS1 + PM2 + PP3
c.1951 + 1G > C	c.1951 + 1G > C	–	–	–	–	–	Pathogenic	PVS1 + PM2 + PP3
c.833T > G	p.Leu278X	–	–	–	–	–	Pathogenic	PVS1 + PM2 + PP4
c.1955G > A	p.W652X	–	–	–	–	–	Pathogenic	PVS1 + PM2 + PP3
c.944_945insCAC	p.P315LinsT	–	–	–	D	–	Uncertain significance	PM2 + PP3
c.1178G > T	p.G393V	D	PD	High	D	33	Uncertain significance	PM2 + PP3
c.1444A > G	p.T482A	D	PD	High	D	25.5	Uncertain significance	PM2 + PM3 + PP3
c.2159G > C	p.R720P	D	PD	Medium	D	33	Uncertain significance	PM2 + PP3

*PD, Probably Damaging; D, Deleterious. The type of evidence refers to. ACMG/AMP 2015 guideline (http://wintervar.wglab.org/).*

## Discussion

Oculocutaneous albinism is a group of autosomal recessive genetic diseases caused by the reduction or lack of melanin synthesis in melanocytes that affects the pigmentation of hair, skin and eyes (Yang et al., 2019). Different types of OCA cannot be completely distinguished by clinical phenotype, therefore, molecular diagnosis have become a useful tool and a necessary condition for genetic consultation (Gronskov et al., 2007). OCA2 is one of the main subtypes of OCA, which is caused by mutations in *OCA2* (Yuasa et al., 2007). The protein encoded by this gene has 12 transmembrane domains and is an intact membrane protein involved in the transport of small molecules, especially tyrosine, which is the precursor of melanin synthesis (Rinchik et al., 1993; Gronskov et al., 2007).

In this study, we collected 28 oculocutaneous albinism patients with compound heterozygous or homozygous *OCA2* variants identified by sequencing. All patients have typical clinical phenotypes of oculocutaneous albinism (Table 1). Thirty one variants have been identified in all patients, including 19 reported variants and 12 novel variants. Among all the variants, missense variants (54.8%, 17/31) were the most common mutation (Chumakov and Kronrod, 1990; Spritz et al., 1997; Sviderskaya et al., 1997; Passmore et al., 1999; Suzuki et al., 2003; Duan et al., 2006; Hongyi et al., 2007; Yuasa et al., 2007; Chiang et al., 2008; Dai et al., 2008; Rooryck et al., 2008; Gronskov et al., 2009; Wei et al., 2010, 2011, 2013; Gargiulo et al., 2011). The missense variants, p.R419W and p.A481T were the most general OCA2 variants, six patients have inherited these variants, respectively. R A Spritz (Spritz et al., 1997) have found p.R419W in OCA2 patients, but the variant classification of the HGMD database and ClinVar database are inconsistent. According to the comprehensive analysis of ACMG, the variant was consistent with uncertain significance. The high frequency of p.R419W in our study could provide more evidence for its pathogenicity. In addition, the pathogenicity of p.A481T has not been determined, it was recorded as benign in the ClinVar database, but reported to be an Asian-specific hypopigmentation allele in 2007 (Yuasa et al., 2007). The p.P743L variant in the OCA2 gene has been reported in the homozygous state or in the compound heterozygous state in multiple unrelated individuals with OCA2 (Lee et al., 1994; King et al., 2003; Hutton and Spritz, 2008; Sengupta et al., 2010; Jaworek et al., 2012; Richards et al., 2015; Shahzad et al., 2017). Variant p.P743L is a semi-conservative amino acid substitution. Due to the differences in physiochemical properties between the two residues, the switch of proline to leucine residue will have significant impact on protein structure, indicating the pathogenic effect of the mutation. In our study, 2 patients inherited this missense substitution, most of the previous reports for this substitution were in European, American and African, this was the first report in Chinese. The splice variant c.1182 + 1G > A was also found in two patients, in 2019, Dan Luo et al., found four patients carrying this mutation, and one of them was homozygous (Luo et al., 2019). In addition, a research showed that the variant was expected to affect splicing following exon 11, thereby leading to abnormal splicing of the transcript (Montoliu et al., 2014).

Among the 31 different mutations in the OCA2 gene, there are 12 novel mutations (4 frameshift, 2 stopgain, 2 splice-site,1 insertion and 3 missense). Frameshift mutations p.R53Gfs^∗^49, p.N279Kfs^∗^17, p.I469Lfs^∗^4, p.I655Nfs^∗^12 and stopgain mutations p.Leu278X, p.W652X were expected to affect the original protein OCA2 function by changing the protein sequence or leading to early termination of protein translation. The truncated protein lacks intact transmembrane domain, which could cause the dysfunction of tyrosine transport and precursor melanin synthesis, and lead to the location of the protein in the nucleus. Splicing mutations c.1637-2A > G and c.1951 + 1G > C were expected to affect splicing of the transcript, thereby leading to abnormal protein function. The literature research showed that most missense mutations occur in the loop between the transmembrane domains (Spritz, 1994). In addition, most of the missense variants found in this study were located in the P_permease domain, which was linked to human melanosomal P gene. Variants in P gene were responsible for classic phenotype of OCA2 (Lee et al., 1995; Puri et al., 2000; Brilliant, 2001; Staleva et al., 2002). Missense variants p.G393V occurred in the fourth transmembrane domain of P protein, which would lead to inactivity of the P protein. Three novel missense variants p.G393V, p.T482A and p.R720P were evolutionary conservation and *in silico* predicted to be damaging or probably damaging. The T482A and R720P are assessed to decrease the OCA2 protein stability, thereby affect to protein function possibly. According to the ACMG, these missense variants are recorded as uncertain significance, therefore, the pathogenicity of these missense mutations needs in-depth exploration.

At present, due to the lack of effective treatment for albinism, prenatal diagnosis is particularly important to prevent the birth of patients. On the basis of prenatal genetic testing and diagnosis, genetic counseling and inspection guidance can effectively prevent the birth of severely patients (Bao et al., 2019; Geng et al., 2019; Li et al., 2019).

In summary, we identified 31 distinct variants of *OC*A2 by next-generation sequencing, in addition, 12 variants were novel ones. We characterized the molecular and phenotypic data for patients with OCA2 variants and revealed one potential missense variant cluster by curating published data. Our findings will benefit not only for the genetic diagnosis and counseling but also provide motivation for further functional characterizations of *OCA2* variants.

## Data Availability Statement

The datasets generated for this study can be found in the China National GeneBank with the accession code of CNP0002229.

## Ethics Statement

Written informed consent was obtained from the individual(s) for the publication of any potentially identifiable images or data included in this article.

## Author Contributions

LM and JZhu analyzed the data and prepared the manuscript. JW and YH collected the data. JZha and CW rechecked the data. DP and YZ provided theoretical guidance. DP revised the manuscript and read and approved the final manuscript. All authors approved the final manuscript.

## Conflict of Interest

JW was employed by Changsha Kingmed Center for Clinical Laboratory. The remaining authors declare that the research was conducted in the absence of any commercial or financial relationships that could be construed as a potential conflict of interest.

## Publisher’s Note

All claims expressed in this article are solely those of the authors and do not necessarily represent those of their affiliated organizations, or those of the publisher, the editors and the reviewers. Any product that may be evaluated in this article, or claim that may be made by its manufacturer, is not guaranteed or endorsed by the publisher.

## Footnotes

1
https://gnomad.broadinstitute.org/

2
http://www.internationalgenome.org

3
http://www.ncbi.nlm.nih.gov/dbvar

4
http://evs.gs.washington.edu/EVS

5
http://www.ncbi.nlm.nih.gov/clinvar

6
http://www.ifpcs.org/albinism

7
http://www.biobase-international.com/product/hgmd

8
https://omim.org/

9
http://sift.jcvi.org

10
http://genetics.bwh.harvard.edu/pph2

11
http://mutationassessor.org/

12
http://provean.jcvi.org/index.php

13
http://cadd.gs.washington.edu/download

14
http://folding.biofold.org/cgi-bin/i-mutant2.0.cgi

15
https://zhanglab.ccmb.med.umich.edu/I-TASSER/

## Supplementary Material

Supplementary Figure 1 Pedigree drawings of the seven families.
