# Supplementary figures and images for: Genetic Analysis of 28 Chinese Families With Tyrosinase-Positive Oculocutaneous Albinism
Source: Front Genet. 2021 Oct 11;12:715437. doi: 10.3389/fgene.2021.715437 (PMC8544823; doi:10.3389/fgene.2021.715437)

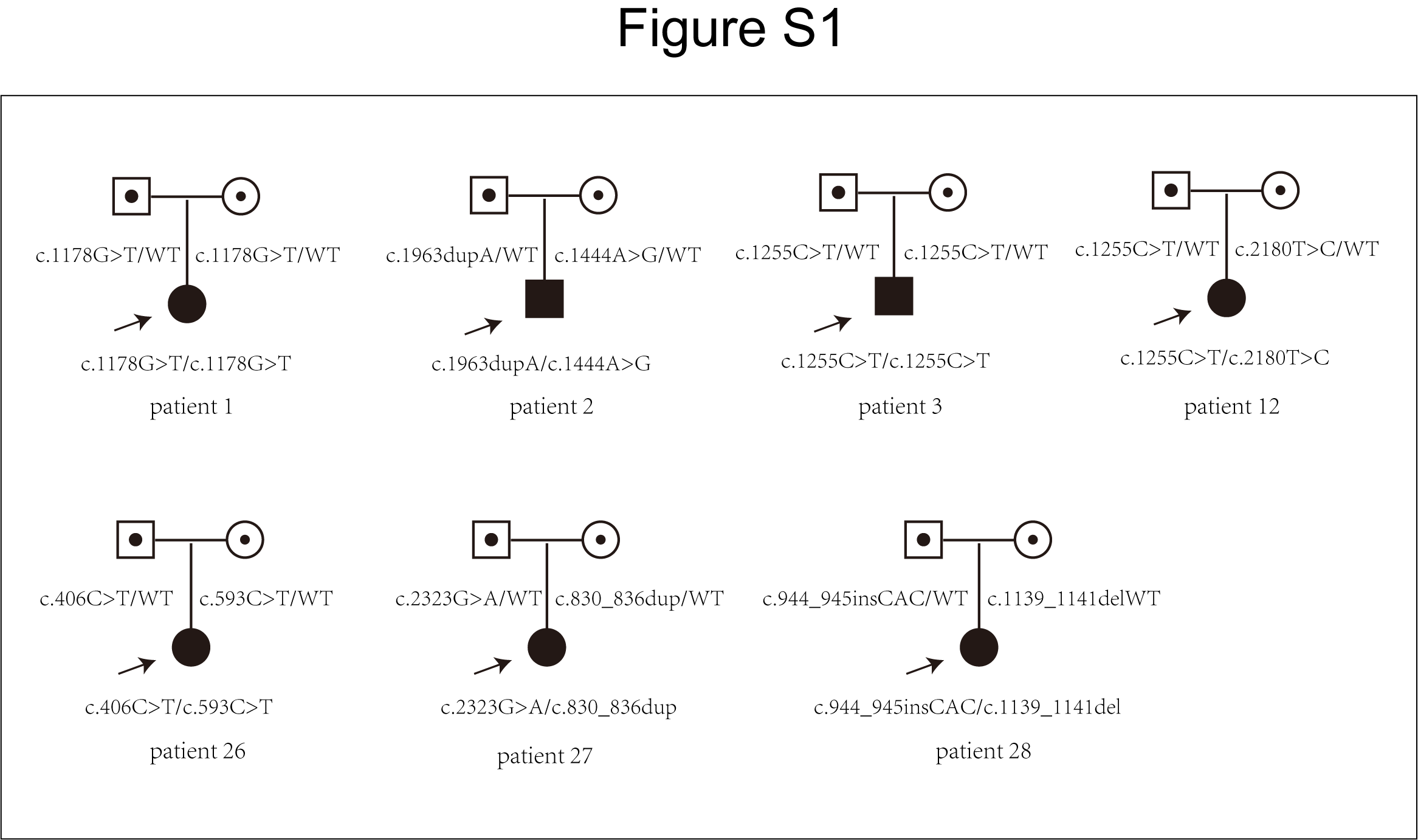

Supplement: Supplementary Figure 1 — Pedigree drawings of the seven families. [file Image_1.TIF]
