# Supplementary material for: Genetic Analysis of 28 Chinese Families With Tyrosinase-Positive Oculocutaneous Albinism
Source: Front Genet. 2021 Oct 11;12:715437. doi: 10.3389/fgene.2021.715437 (PMC8544823; doi:10.3389/fgene.2021.715437)
Supplement: Supplementary file 2 [file Table_1.pdf]

Table S1. Primers used in PCR for amplification of *OCA2* gene

Nt change	Forward	Reverse	Size (bp)
c.944_945insCAC	CTGTATGGTTCCCTTTCTACCTAGA	GCTCTTCCAGCTGTGAGATTG	460
c.830_836dup	TCCAAGTCACATGCTGACCT	GACCGAGCAGTGCCAGAT	278
c.593C>T	CTCATCAGACATCCTGGAGAA	GCGTACTGGATGCACACAGTA	339
c.406C>T	GCCTACCTCAGCTTGGAAAGA	GAAAGACCAGGGTTGATTCG	307
c.2323G>A	CGACCCACACAGGCACA	TGGTGGCAGCTGGCAT	583
c.2180T>C	CTACACCTGTGAGTGCAGCA	TTGCACACCTATGTCTGCCT	368
c.1963dupA	CTCTCAGTGGCTAAGGTAAAGCT	GAGGTACAAGAACATAGGCATGAA	548
c.1444A>G	TCTAGGATTGAAGGACCAGTCAC	AGGCTCACTCTGGAAAGGAA	533
c.1255C>T	ATGTCTCCCCATGAGACCAC	AGGTTGTCTAACCAGAATACCGAT	725
c.1178G>T	GAGATTCATGAGACCTGCACTA	CATTAGGCTCATCACTGGGAT	278
c.1139_1141del	CCTCAGGAGATTCATGAGACCT	AGTGGATGGTGAGATTTCCAA	350
